# Laminar Flow Protects Vascular Endothelial Tight Junctions and Barrier Function via Maintaining the Expression of Long Non-coding RNA MALAT1

**DOI:** 10.3389/fbioe.2020.00647

**Published:** 2020-06-25

**Authors:** Fangfang Yang, Yunpeng Zhang, Juanjuan Zhu, Jin Wang, Zhitong Jiang, Chuanrong Zhao, Qianru Yang, Yu Huang, Weijuan Yao, Wei Pang, Lili Han, Jing Zhou

**Affiliations:** ^1^Department of Physiology and Pathophysiology, School of Basic Medical Sciences, Peking University, Beijing, China; ^2^Key Laboratory of Molecular Cardiovascular Science, Ministry of Education, Beijing, China; ^3^NHC Key Laboratory of Cardiovascular Molecular Biology and Regulatory Peptides, Beijing, China; ^4^Shenzhen Research Institute, Institute of Vascular Medicine and Li Ka Shing Institute of Health Sciences, Chinese University of Hong Kong, Hong Kong, China

**Keywords:** endothelial permeability, shear stress, MALAT1, Nesprins, β-catenin, endothelial cells

## Abstract

Atherosclerotic plaque preferentially develops in arterial curvatures and branching regions, where endothelial cells constantly experience disturbed blood flow. By contrast, the straight arteries are generally protected from plaque formation due to exposure of endothelial cells to vaso-protective laminar blood flow. However, the role of flow patterns on endothelial barrier function remains largely unclear. This study aimed to investigate new mechanisms underlying the blood flow pattern-regulated endothelial integrity. Exposure of human endothelial cells to pulsatile shear (PS, mimicking the laminar flow) compared to oscillatory shear (OS, mimicking the disturbed flow) increased the expressions of long non-coding RNA MALAT1 and tight junction proteins ZO1 and Occludin. This increase was abolished by knocking down MALAT1 or Nesprin1 and 2. PS promoted the association between Nesprin1 and SUN2 at the nuclear envelopes, and induced a nuclear translocation of β-catenin, likely through enhancing the interaction between β-catenin and Nesprin1. In the *in vivo* study, mice were treated via intraperitoneal injection with β-catenin agonist SKL2001 or its inhibitor XAV939, and they were then subjected to Evans blue injection to assess aortic endothelial permeability. The aortas exhibited a reduced wall permeability to Evans blue in SKL2001-treated mice whereas an enhanced permeability in XAV939-treated mice. We concluded that laminar flow promotes nuclear localization of Nesprins, which facilitates the nuclear access of β-catenin to stimulate MALAT1 transcription, resulting in increased expressions of ZO1 and Occludin to protect endothelial barrier function.

## Introduction

Hemodynamic forces are the pivotal modulators of both physiological and pathological behaviors of vascular endothelia cells (ECs) and atherogenesis. Clinical evidence show that atherosclerotic lesions occur primarily at the curvature and bifurcations of arteries, where ECs are exposed to disturbed blood flow with low and oscillatory shear (OS). OS turns vascular ECs toward a more active phenotype with reduced expressions of genes related to endothelial integrity and high permeability to circulating white blood cells and lipids (Chiu and Chien, 2011; Zhou et al., 2014). By contrast, the straight arteries are relatively protected from development of atherosclerosis due to the beneficial effect of laminar blood flow with unidirectional and pulsatile shear (PS) on the endothelial barrier function (Chiu and Chien, 2011). Recent studies showed that long non-coding RNAs (lncRNAs) participate in flow patterns-induced regulation of endothelial function (Huang et al., 2017). Although several lncRNAs have been found to associate with ECs function, including metastasis-associated lung adenocarcinoma transcript 1 (MALAT1) that was reported to regulate ECs proliferation and migration (Michalik et al., 2014), the involvement of lncRNAs in blood flow-regulated endothelial barrier function is basically unknown.

LncRNAs are commonly defined as ncRNA molecules with lengths exceeding 200 nucleotides. They are involved widely in molecular and cellular functions via distinct mechanisms that are only partially understood (Wilusz et al., 2009). MALAT1 is a highly conserved lncRNA and broadly expressed in tissues including ECs of both macro- and microvasculatures (Michalik et al., 2014). MALAT1 contributes to the maintenance of blood-tumor barrier (Ma et al., 2016) and blood-brain barrier (Ruan et al., 2019) probably through regulating the expressions of endothelial tight junction proteins, including Zonula Occludens 1 (ZO1) and Occludin (Ma et al., 2016; Ruan et al., 2019). These studies suggest that MALAT1 protects endothelial integrity. However, it is yet to determine whether MALAT1 participates in flow-regulated endothelial barrier function.

In cultured podocytes, β-catenin was shown to activate the transcription of MALAT1 by its nuclear translocation and subsequent binding to the promoter region of MALAT1 (Hu et al., 2017). This study prompted us to postulate a positive correlation between β-catenin signaling and endothelial barrier function. Particularly, β-catenin was found to be essential for blood-brain barrier development and maintenance in endothelial integrity in the microvasculature (Benz et al., 2019; Laksitorini et al., 2019). Activation of the Wnt/β-catenin axis by exogenous Wnt ligand improved the blood-brain barrier phenotype in culture cell model and reduced the paracellular permeability (Laksitorini et al., 2019). Nevertheless, the mechanisms linking Wnt/β-catenin activation to arterial endothelial barrier function are still lacking.

The linker of nucleoskeleton and cytoskeleton (LINC) complex, primarily composed of Nesprins (nuclear envelope spectrin repeats), SUNs (Sad1p-UNC-84), and lamins, is a proposed mechanical bridge tethering the nucleoskeleton and cytoskeleton via the nuclear envelope (Padmakumar et al., 2005; Crisp et al., 2006; Haque et al., 2006). The largest isoforms of mammalian Nesprins (Nesprin1 and Nesprin2) can directly associate with the LINC protein SUNs on the inner nuclear membrane; SUNs, in turn, bind to lamins on the nuclear scaffold (Wang et al., 2009). Cytoplasmic domains of Nesprins on the outer nuclear membrane link with actin filaments, microtubules and intermediate filaments (Alam et al., 2016). Nesprins and SUNs are thereby forming a physical linkage between outer and inner nuclear membranes, allowing direct transmission of mechanical signals. Defects in the Nesprin-SUN link lead to impaired nuclear mechanotransduction (Uhler and Shivashankar, 2017). β-catenin lacks classical nuclear localization signals, but may interact with the nuclear pore complex (NPC) directly on the surface of the nuclear membrane to mediate β-catenin transfer to the nucleus (Jamieson et al., 2014). Interestingly, the LINC complex was found to regulate nuclear accumulation ofβ-catenin, probably through a direct interaction between β-catenin and Nesprins or SUNs (Zhang et al., 2016; Uzer et al., 2018). The LINC complex can provide anchors to locate β-catenin on the surface of the nucleus next to the NPC, thereby localizing its inward metastasis (Uzer et al., 2018).

In this study, we revealed the role and the underlying mechanism of PS in protecting vascular endothelial tight junction and barrier function. We found that exposure of endothelial cells to PS, but not OS, increased the expressions of tight junction proteins ZO1 and Occludin, together with long non-coding RNA, MALAT1; these effects were reversed by knocking down MALAT1 with siRNAs. The upregulation of MALAT1, ZO1 and Occludin by PS could be suppressed by knocking down nesprin1 and nesprin2. PS enhanced the colocalization and association of nesprin1 with SUN2 at the nuclear envelopes and caused a nuclear translocation of β-catenin; the latter is likely through enhancing the physical interaction between β-catenin and nesprin1. We also provide *in vivo* evidence showing that SKL2001 (an agonist of the Wnt/β-catenin pathway) reduced arterial wall permeability whereas XAV939 (an inhibitor of the Wnt/β-catenin pathway) enhanced the permeability. The present study provide new evidence that PS protects endothelial tight junctions and barrier function through maintaining the expression of MALAT1 and that PS promotes the interaction between nesprin1 and β-catenin to facilitate a nuclear translocation of β-catenin to increase MALAT1 expression.

## Materials and Methods

### Cell Culture and Treatments

Primary human umbilical vein endothelial cells (HUVECs) were isolated and cultured in Medium 199 supplemented with 10% fetal bovine serum (FBS) (Gemini 900-008), 4 ug/mL of endothelial cell growth factor (ECGF) (Sigma E1388), 1% penicillin/streptomycin, at 37°C with 5% CO_2_. Cells at passages 5–7 were used for all *in vitro* experiments. For inhibition or activation of the Wnt/β-catenin pathway, cells were either incubated with XAV939 (Selleck S1180) or SKL2001 (Selleck S8320) in culture medium at a concentration of 20 or 30 μmol/L, respectively, for 12 h at 37°C with 5% CO_2_.

### Shear Experiment

A parallel plate apparatus was used in this study to apply fluid shear stress to HUVECs through liquid flow. Shear stress (τ) on the cell monolayer in the flow chamber was calculated using the momentum balance for a Newtonian fluid and assuming chamber geometry (Frangos et al., 1988): τ = 6 Qμ/(wh^2^), where Q is the flow rate (mL/min); μ is perfusion viscosity (0.0069 dynes second/cm^2^); h is the channel height (0.025 cm); w is the slit width (5 cm); and τ is the wall shear stress (dynes/cm^2^). The flow rate was controlled by adjusting the relative distance between the two reservoirs by changing the length of the overflow manifold tubing. The flow rates were monitored by an electromagnetic flow probe. The system was kept in a constant-temperature controlled enclosure, with pH maintained at 7.4 by continuous gassing with a humidified mixture of 5% CO_2_ in air. Cells were seeded on a collagen (50 μg/mL) coated glass plate and were placed in the flow chambers to be exposed to PS (12 ± 4 dynes/cm^2^) or OS (0.5 ± 4 dynes/cm^2^). The flow with PS or OS is composed of a mean flow with shear stress at 12 or 0.5 dynes/cm^2^ supplied by a hydrostatic flow system to provide the basal nutrient and oxygen delivery, and the superimposition of a sinusoidal oscillation using a piston pump with a frequency of 1 Hz and a peak-to-peak amplitude of ±4 dynes/cm^2^. For applying PS (12 ± 4 dynes/cm^2^) to the cell monolayer, a flow rate of 54.3 mL/min was utilized; while for applying OS (0.5 ± 4 dynes/cm^2^), the flow rate was set approximately to be 2.3 mL/min.

### RNA Isolation and Quantitative RT-PCR

Total RNA from HUVECs was extracted with Trizol reagent (Applygen R1030). The isolated RNA was reverse-transcribed into complementary DNA using the Oligo (dT) primers with the M-MLV RT system (Promega M1705). Real-time PCR samples were prepared by mixing cDNAs, power-SYBR Mix (Yeasen 11202ES08) and specific primer sets (Table S1). The initial denaturation step of PCR amplification was 95°C for 10 min, followed by 40 cycles of 95°C for 15 s and 55°C for 1 min, then 95°C for 15 s and 60°C for 1 min, and last at 95°C for 1 s. Gene expressions were normalized against GAPDH.

### Western Blot Assay

Cells were lysed with RIPA lysis buffer: 25 mmol/L HEPES, pH 7.4, 1% Triton X-100, 1% deoxycholate, 0.1% SDS, 125 mmol/L NaCl, 5 mmol/L EDTA, 50 mmol/L NaF, 1 mmol/L PMSF. Equal amounts of protein were separated on SDS-PAGE, transferred to nitrocellulose membranes, blocked in 5% skimmed milk in TBS containing 0.1% Tween 20, and incubated with the primary antibodies against ZO1 (1:1,000, Proteintech 21773-1-AP), Occludin (1:1,000, Proteintech 13409-1-AP), β-catenin (1:1,000, Abclonal A10834), Tubulin (1:1,000, Proteintech 10094-1-AP), Histone-H3 (1:1,000, Proteintech 17168-1-AP), or GAPDH (1:1,000, Easybio BE0024) overnight at 4°C, followed by detection with donkey anti-rabbit/-mouse/-goat IgG (Abcam) antibody IRDye 800/700 Conjugated. Visualization was performed with an Odyssey infrared imaging system (LI-COR Biosciences).

### Immunofluorescence

HUVECs on glasses were fixed with 4% paraformaldehyde for 20 min, permeabilized with cold phosphate buffered saline (PBS) containing 0.01% Triton X-100 for 10 min, incubated with blocking buffer (3% bovine serum albumin in PBS) for 60 min, and then incubated overnight with primary antibodies against ZO1 (1:50), Occludin (1:30), β-catenin (1:100), Nesprin1 (1:100, Huabio ET7107-28), SUN2 (1:100, Millipore MABT880). The cells were washed with PBS, and incubated with secondary antibody, such as Alexa Fluor 488-conjugated goat anti-rabbit/-mouse IgG (1:500, Abcam ab150077/150113) or Alexa Fluor 555-conjugated goat anti-rabbit/-mouse IgG (1:500, Abcam ab150078/ab150114). The nuclei were stained with DAPI. The mounted slides or slips were visualized by fluorescence microscopy (Leica DMI6000B or Leica TCS SP8).

### Proximity Ligation Assay (PLA)

Duolink *in situ* PLA was performed according to the protocol from the manufacturer (Sigma-Aldrich). In brief, HUVECs on glasses were fixed with 4% paraformaldehyde for 10 min at room temperature, washed with PBS and permeabilized with 0.5% Saponin for another 10 min at room temperature. Unspecific binding sites for antibodies were blocked for 30 min with blocking buffer at 37°C. The samples are incubated with primary antibodies SUN2 (1:100) and Nesprin1 (1:100) overnight at 4°C. On the following day, samples were washed with Buffer A (0.01 mol/L Tris, 0.15 mol/L NaCl and 0.05% Tween 20). Secondary antibodies conjugated with oligonucleotides (PLA probe MINUS and PLA probe PLUS) were added into the reaction and the mixtures were incubated for 60 min at 37°C. Samples were washed with buffer A and incubated with ligation solution for 30 min at 37°C. The Ligation solution consists of two oligonucleotides (illustrated as red bands) and ligase. Samples were then washed with buffer A and were incubated with amplification solution for 100 min at 37°C. The Amplification solution consists of nucleotides, fluorescently labeled oligonucleotides, and polymerase. The oligonucleotide arm of one of the PLA probes acts as a primer for a rolling-circle amplification (RCA) reaction using the ligated circle as a template, generating a concatemeric (repeated sequence) product. Afterword the samples were washed with buffer B (0.2 mol/L Tris and 0.1 mol/L NaCl), stained with DAPI to indicate the nuclei, and analyzed by fluorescence microscopy.

### Cell Permeability Assay

HUVECs were grown on polycarbonate membrane of transwell with 0.4 μm pores. Six hundred microliters of cell culture medium was placed in the lower chamber (24-well plate) of a transwell, and 200 μl of the culture medium containing 20 nmol/L dextran (Sigma FD40S) was placed in the upper chamber. They were placed in a 37° incubator containing 5% CO_2_ for 1 h. Medium in the lower chamber was removed, diluted three times with PBS, and was measured for fluorescence value with a microplate reader. Meanwhile, the polycarbonate membrane in the transwell was carefully removed with a needle, cells on the membrane were stained with DAPI, and the number of nuclei was counted under a fluorescence microscope.

### Extraction of Nuclear Proteins

HUVECs were scraped and lysed with Buffer A (10 mmol/L Tris pH7.5, 1.5 mmol/L MgCl_2_, 10 mmol/L KCL, 0.5% NP-40). Nuclei were precipitated by centrifugation at 7,000 rpm for 5 min. The supernatants were collected and stored for further experiments (containing cytoplasmic and membrane proteins). Nuclei proteins were extracted at 4°C by gentle resuspension with buffer B (2× volume of the pallet, 20 mmol/L Tris (pH7.5), 1.5 mmol/L MgCl_2_, 420 mmol/L NaCL, 10% Glycerol, 0.2 mmol/L EDTA), followed by incubation on a platform for 30 min. The mixtures were centrifugated at 13,000 rpm for 15 min to obtain the nuclei proteins.

### Co-immunoprecipitation Assay

Cells were trypsinized and lysed with Lysis Buffer containing 25 mmol/L Tris-HCl pH 7.4, 150 mmol/L NaCl, 0.5% NP-40, 0.5% sodium deoxycholate, 5 mmol/L EDTA and a protease inhibitor Cocktail (Baidiebio De5002). Protein concentration of the lysate was adjusted to be 2–3 μg/μL. 1–1.5 μg of antibody against β-catenin or control IgG were added into 100 μl of cell lysate, followed by an overnight incubation at 4°C on a shaker. Protein A or G beads (Santa sc-2003) were added and incubated for 1–4 h at 4°C to pull down the immune complexes. The beads were washed with lysate for at least three times. 50 μl of 2 × SDS buffer were added into each sample, which was then subjected to Western blot analysis.

### *En face* Analysis of Aortic Endothelium

C57BL/6 wild-type mice (12 weeks old, 22–25 g) were anesthetized, and were fixed for 5 min by perfusion through left cardiac ventricle with 4% paraformaldehyde in PBS buffer under physiological pressure. Aortas were harvested, fixed with 4% paraformaldehyde in PBS buffer for no more than 1 day, and were then longitudinally dissected with microdissecting scissors and pinned flatly on a black wax dissection pan. The luminal surfaces of the aortas were exposed, blocked with 3% bovine serum albumin in PBS for 1 h at room temperature, and were incubated with primary antibodies against ZO1 (1:50), CD31 (1:100) or Occludin (1:30) at 4°C overnight. The aortas were washed three times with PBS, and were then probed with secondary antibodies including Alexa Fluor 488-conjugated goat anti-rabbit/-mouse IgG (1:500) or Alexa Fluor 555-conjugated goat anti-rabbit/-mouse IgG (1:500). Nuclei were counterstained with DAPI for 5 min at room temperature. Image acquisition was performed using a confocal microscope.

### Assay for Endothelial Permeability to Evans Blue

C57BL/6 mice were intraperitoneally injected with a β-catenin inhibitor XAV939 (4 mg/kg in 100 μl of DMSO) or a β-catenin activator SKL2001 (6 mg/kg in 100 μl of DMSO) for 7 days. Control groups were injected with DMSO. Evans blue dye (30 mg/kg in 100 ul PBS) was injected into the tail vein of the mice with an insulin needle at a rate of 0.01 mL/g of animal weight. Once finished injection, remove the needle and press the injection site with cotton to stop bleeding. After 30 min of injection, the mice were euthanatized, perfused with PBS, and the aortas were quickly removed. The aortas were photographed with a camera to observe the leakage of dye. The aorta arches were then dissected, weighted, and incubated in 1 mL of formamide overnight (or 2 days) at 55°C. The Evans blue dye was extracted from aorta arch with formamide and was measured spectophotometrically at 620 nm to obtain the absorbance according to a standard curve. Vascular endothelial permeability was calculated as the weight of Evans blue dye in one gram of aortas.

### siRNA-Mediated Gene Silencing

HUVECs grown at 80% confluence were transfected with 40 nmol/L siRNA-MALAT1, 60 nmol/L siRNA-Nesprin1, 60 nmol/L siRNA-Nesprin2 and siRNA-Control using Lipofectamine 2000 (Invitrogen 11668019) to knock down the expressions of the indicated genes. After transfection, the cells were starved for 6 h in reduced serum medium (Opti-MEM) (invitrogen, 31985070) at 37°C with 5% CO_2_. The culture media were discarded and replaced with fresh culture media of endothelial cells without growth factors and containing 5% serum. The cells were further cultured for 24 h. Sequence of the siRNAs are presented in Table S2.

### Statistical Analysis

Data are expressed as mean ± SEM from at least three independent experiments. Statistical analysis was performed by unpaired or paired *t*-test for 2 groups of data and by one-way ANOVA for multiple comparisons. Statistical significance among multiple groups was determined by *post-hoc* analysis (Tukey honestly significant difference test). Values of *P* < 0.05 were considered statistically significant.

## Results

### PS Protects Endothelial Barrier Function and Maintains the Expressions of the Tight Junction Proteins ZO1 and Occludin

**Figure 1:**
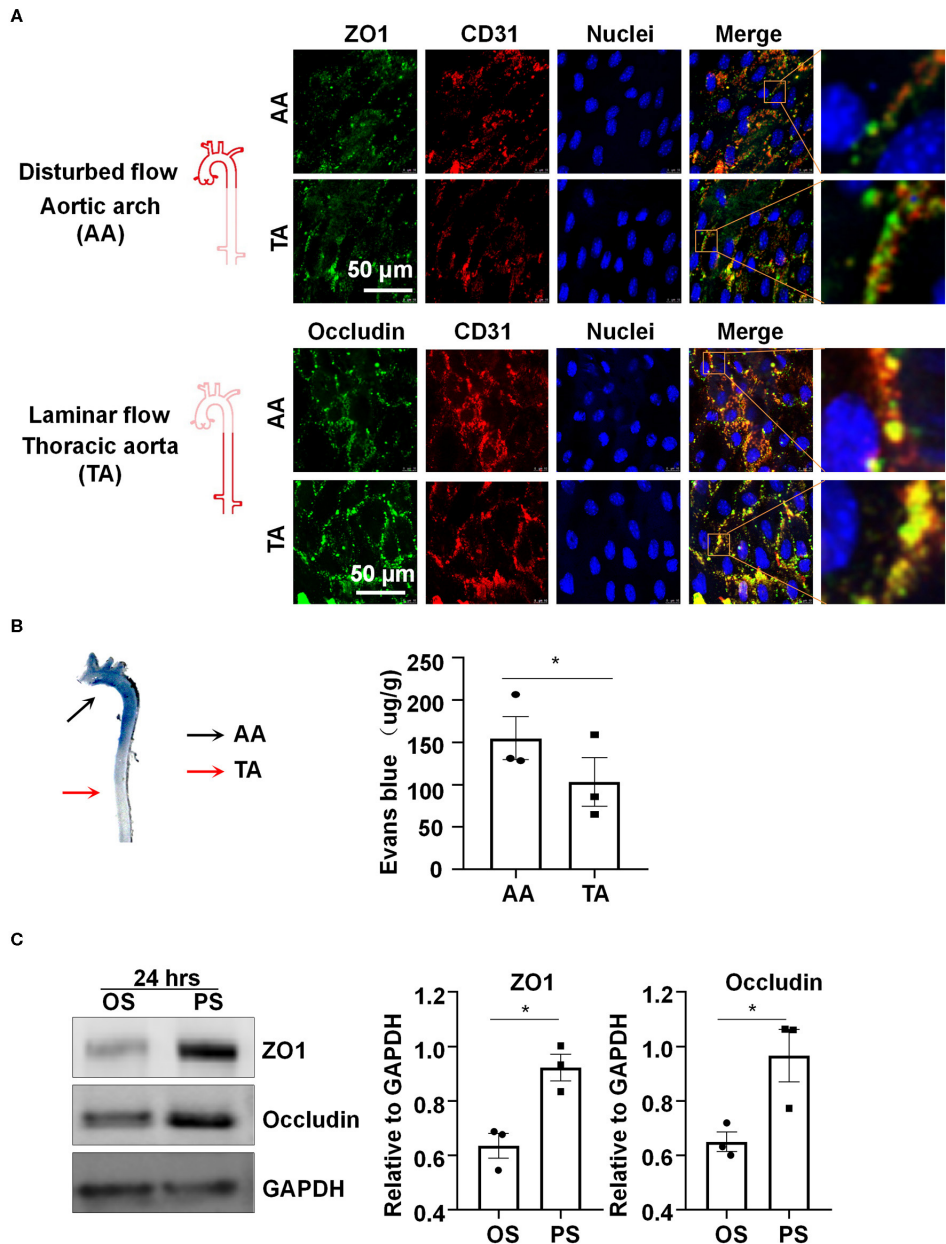
Laminar blood flow with uniform and pulsatile shear (PS) protects the endothelial barrier function and maintain the expressions of the tight junction proteins ZO1 and Occludin. **(A)** Cellular localizations of ZO1 (green), Occludin (green), and CD31 (red) from the inner curvature of the aortic arch (AA) or from the thoracic aorta (TA) of C57BL/6 wild type mice were assessed by en face immunofluorescence. Images are representatives of 3–5 independent experiments with similar results. **(B)** Evans blue (30 mg/kg in 100 ul PBS) was injected into tail vein of mice with an insulin needle, and the aortas were isolated for photographing to observe the leakage of Evans blue in the aortic wall. Evans blue dye was extracted from aorta arch with formamide and measured spectophotometrically at 620 nm to determine endothelial permeability. Each symbol represents one animal. Paired *t*-test. *P < 0.05 compared with the indicated controls. **(C)** HUVECs were exposed to pulsatile shear (PS, 12 ± 4 dynes/cm^2^) or oscillatory shear (OS, 0.5 ± 4 dynes/cm^2^) for 24 h, and the expressions of ZO1 and Occludin were analyzed by Western blot. Semi-quantification of the blots are shown in the right panels. Unpaired *t*-test. *P < 0.05 compared with the indicated controls.

Platelet EC adhesion molecule-1 (PECAM-1/CD31) is a cell-cell adhesion molecule expressed at the intercellular junction of vascular ECs. In the mouse aorta, co-localization of ZO1 or Occludin with CD31 at endothelial cell-to-cell junctions was low and discontinuous in the inner curvature of aortic arch (AA), where blood flow is disturbed (Figure 1A). By contrast, co-localization was much greater and ZO1 or Occludin distributed fluently along cell-to-cell junctions in the descending thoracic aorta (TA), where blood flow is mostly laminar (Figure 1A). These results indicate that laminar flow with uniform and PS may be required to maintain endothelial barrier integrity, which was further supported by reduced endothelial permeability to Evans blue dye (Figure 1B) in the athero-protective TA as compared to the athero-prone AA.

In order to study the regulation of shear stress with distinct flow patterns on the expressions of tight junction proteins *in vitro*, cultured human ECs were subjected to OS at 0.5 ± 4 dynes/cm^2^ or PS at 12 ± 4 dynes/cm^2^ for 24 h, on a parallel-plate flow system. Western blot assay showed upregulation of ZO1 and Occludin after exposure to PS as compared to OS (Figure 1C). The results also reveal that PS, but not OS, could maintain the expressions of endothelial tight junction proteins.

### PS Upregulates Long Non-coding RNA MALAT1 to Maintain Endothelial Barrier Function

**Figure 2:**
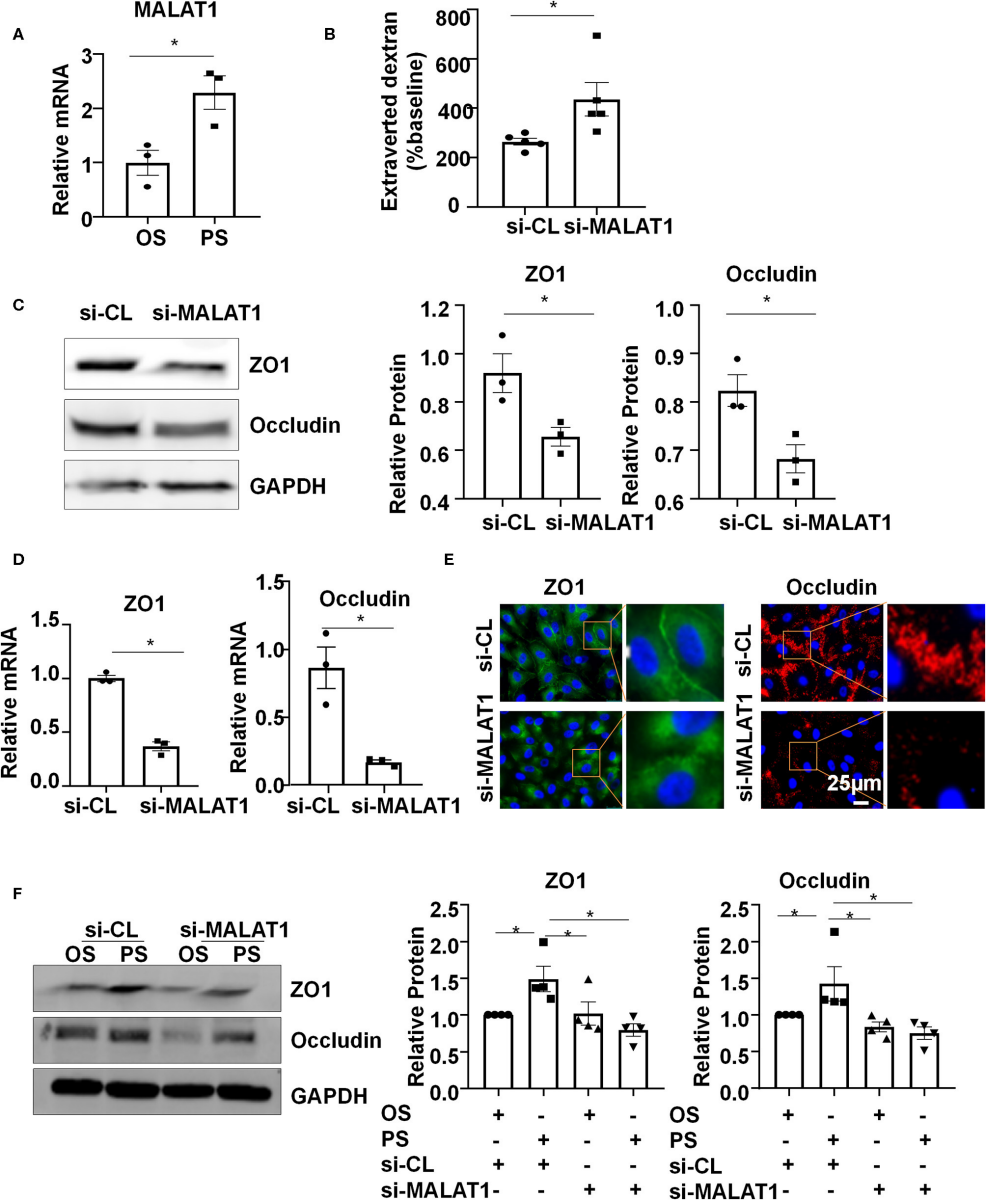
Pulsatile shear (PS) upregulates long non-coding RNA MALAT1 to maintain the endothelial barrier function. **(A)** HUVECs were exposed to PS or OS for 6 h, and the expression of MALAT1 was analyzed by qRT-PCR. **(B)** HUVECs were seeded on polycarbonate membrane in transwells, transfected with siRNAs targeting MALAT1 (si-MALAT1) or control siRNA (si-CL), and endothelium permeability was measured by assaying the extravasation of 40-kDa dextran in the culture medium of the lower chamber. **(C–E)** HUVECs were transfected with si-MALAT1 (40 nmol/L) or si-CL, and the expressions and cellular localizations of ZO1 and Occludin were analyzed by Western blot **(C)**, qRT-PCR **(D)**, and immunofluorescence staining **(E)**. In **(C)**, semi-quantification of the blots are shown in the right panels. **(F)** HUVECs were transfected with si-MALAT1 or si-CL and were then exposed to PS or OS for 6 h, and the expressions of ZO1 and Occludin were analyzed by Western blot. Semi-quantification of the blots are shown in the right panels. Statistical analysis of **(A–D)** were performed by unpaired *t*-test, **(F)** was performed by One-way ANOVA, *P < 0.05 compared with the indicated controls.

Six-hour PS markedly increased the expression of MALAT1, compared with the application of OS (Figure 2A). To understand the functional consequence of the MALAT1 upregulation, we knocked down the expression of MALAT1 in ECs with siRNAs (Figure S1) and measured endothelium permeability by assaying extravasation of 40-kDa dextran. Notably, MALAT1 knockdown increased extravasated dextran compared with scramble control (Figure 2B). The number between si-MALAT1 and si-CL transfected cells was comparable after 24 h post-transfection (Figure S2). The increase in extravasated dextran is probably caused by the regulation of MALAT1 on the expressions of ZO1 and Occludin, as evidenced by the Western blot and quantitative RT-PCR results of down-regulation of both protein and mRNA levels of ZO1 and Occludin in cells transfected with si-MALAT1 (Figures 2C,D). The distribution of tight junction proteins ZO1 and Occludin along the cell-to-cell junctions became disrupted in the si-MALAT1 transfected cells (Figure 2E), whereas the cell adherences junctions exhibited no apparent difference between cells with si-MALAT1 and si-CL transfection, as indicated by the immunofluorescent analysis of adherences junction protein VE-cadherin (Figure S3). Taken together, these results suggest that MALAT1 may be required for the expression of tight junction proteins ZO1 and Occludin to maintain endothelial barrier function.

To investigate whether PS (vs. OS) upregulates the expressions of ZO1 and Occludin via increasing MALAT1, ECs were transfected with si-MALAT1 or si-CL and were then subjected to OS or PS for 24 h. In the si-CL transfected cells, PS promoted the ZO1 and Occludin expressions (Figure 2F). Knocking down MALAT1 reduced the PS-induced upregulation of ZO1 and Occludin while it further reduced their expressions in OS-exposed cells (Figure 2F). These results indicate that PS could induce MALAT1 expression to maintain the expressions of tight junction proteins and endothelial integrity.

### Nesprin1 and Nesprin2 Are Required for Upregulating the Expressions of MALAT1, ZO1, and Occludin by PS

**Figure 3:**
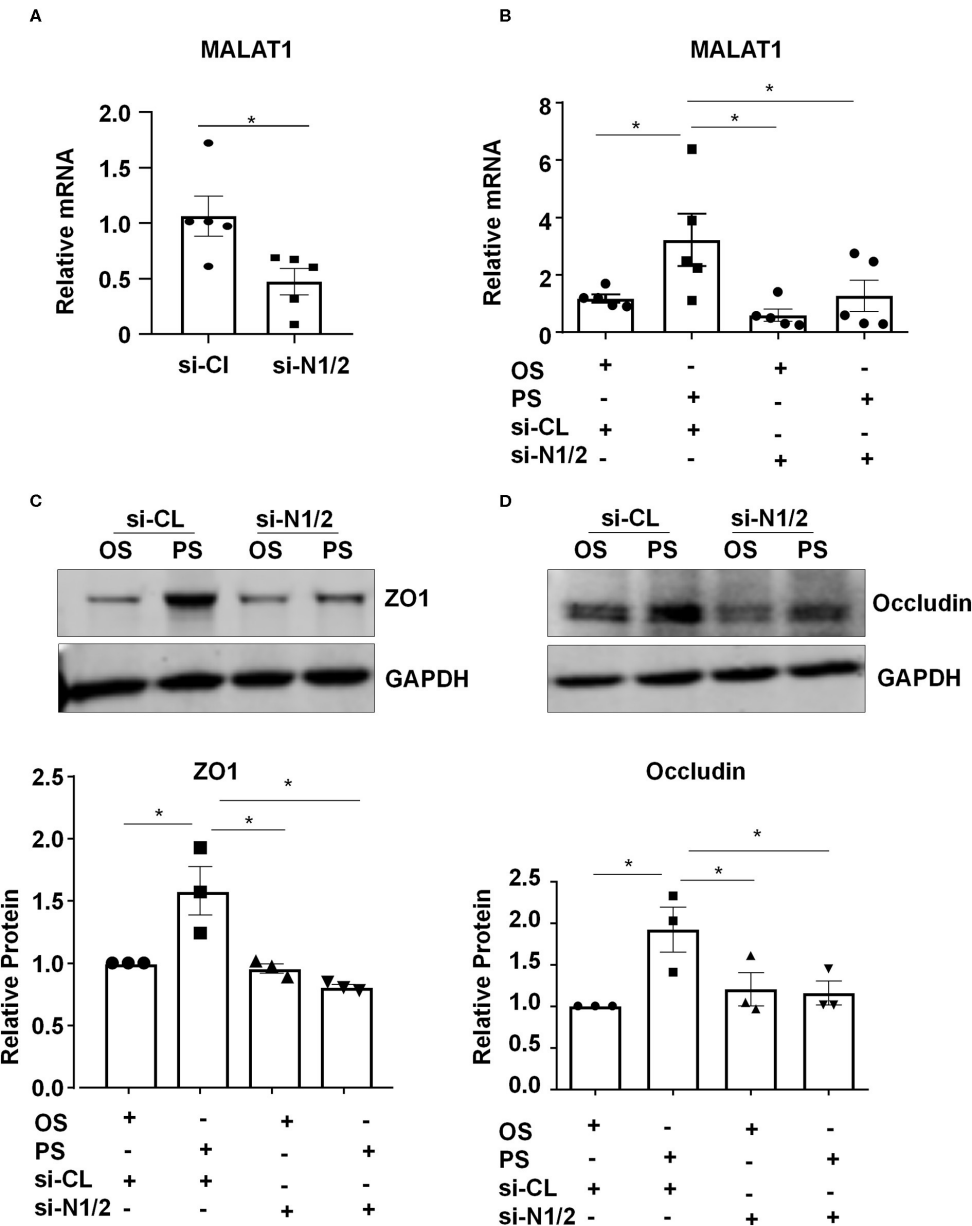
Components of the linker of nucleoskeleton and cytoskeleton (LINC) complex, Nesprin1 and Nesprin2, are required for upregulating the expression of MALAT1, ZO1, and Occludin by pulsatile shear (PS). **(A)** HUVECs were transfected with siRNAs targeting Nesprin1 and Nesprin2 (si-N1/2) (60 nmol/L) or control siRNA (si-CL), and the expression of MALAT1 was analyzed by qRT-PCR. **(B–D)** HUVECs were transfected with si-N1/2 or si-CL and were then exposed to PS or OS for 6 h, and the expressions of MALAT1, ZO1 and Occludin were analyzed by qRT-PCR and Western blot. In **(C,D)**, semi-quantification of the blots are shown in the lower panels. One-way ANOVA, *P < 0.05 compared with the indicated controls.

We investigated whether Nesprins-dependent mechanotranduction is required for the shear-induced regulation of expressions of MALAT1 and tight junction proteins. First, ECs were transfected with siRNAs targeting Nesprin1 and Nesprin2 (si-N1/2) or si-CL under a static condition (Figure S4). The expression of MALAT1 in si-N1/2 transfected cells was decreased to 55.4% of si-CL-transfected cells (Figure 3A). Next, ECs were transfected with si-N1/2 or si-CL and were then subjected to OS or PS for 6 h (for detecting MALAT1) or 24 h (for detecting ZO1 and Occludin). The MALAT1 expression as well as the protein levels of ZO1 and Occludin were induced by PS; however, the induction was attenuated or abolished by knockdown of Nesprin1/2 (Figures 3B–D). These results suggest that Nesprins, the mechanical bridge tethering the nucleoskeleton and cytoskeleton, are necessary for PS-maintained endothelial integrity.

### PS Reinforces the Nesprins-Dependent Cytoplasmic- and Nuclear-Skeleton Link to Facilitate the Nuclear Access of β-Catenin

**Figure 4:**
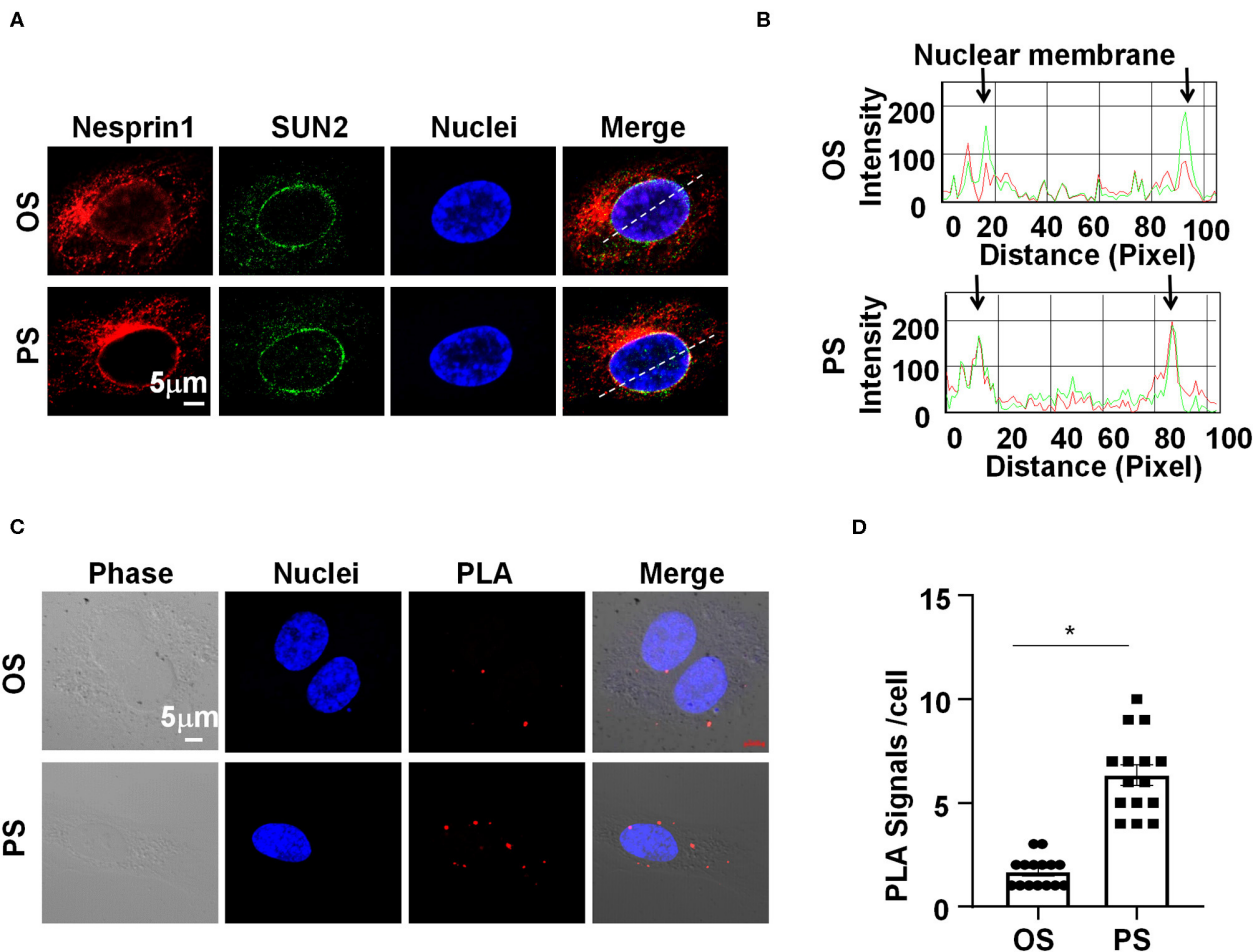
Pulsatile shear (PS) reinforces the Nesprins-dependent cytoplasmic- and nuclear-skeleton link at the nuclear envelope. **(A)** HUVECs were exposed to PS or OS for 6 h, and the localizations of Nesprin1 and SUN2 were analyzed by Immunofluorescence staining. **(B)** Fluorescence intensity profiles of **(A)** analyzed using Image-Pro Plus indicate the degree of overlap between Nesprin1 and SUN2. **(C)** HUVECs were exposed to PS or OS for 6 h, and the association between Nesprin1 and SUN2 was analyzed by PLA. **(D)** Quantification of **(C)**. Each symbol represents one cell. Unpaired *t*-test. *P < 0.05 compared with the indicated controls.

We sought to determine how Nesprins modulate the expression of MALAT1. Since Nesprin1 and Nesprin2 share a similar function to mediate nuclear positioning via microtubule motors and actin (Morgan et al., 2011; King et al., 2014), our study mainly focused on Nesprin1. Nesprin1 can bind to SUN2 to form an important bridge connecting the cytoplasmic skeleton with the nuclear skeleton (Wang et al., 2009). The association between Nesprin1 and SUN2 in ECs was suggested by immunofluorescent staining followed by fluorescence intensity profiling (Figures 4A,B). Some of the Nesprin1 signals were co-localized with SUN2 at the nuclear envelope in ECs exposed to OS, whereas this co-localization can be greatly enhanced by PS (Figures 4A,B). It is of note that the Nesprin1 distribution at the nuclear envelope in ECs exposed to PS was more continuous than that in OS-exposed cells (Figure 4A). Enhanced interaction between Nesprin1 and SUN2 in PS-exposed ECs was confirmed by *in situ* PLA assay showing the punctuated signals indicative of a close association of these two proteins was increased from 2 ± 1 per cell in OS to 7±3 per cell in PS (Figures 4C,D). The findings indicate that PS potentially reinforces the mechanical link between cytoplasmic skeleton with nuclear skeleton.

**Figure 5:**
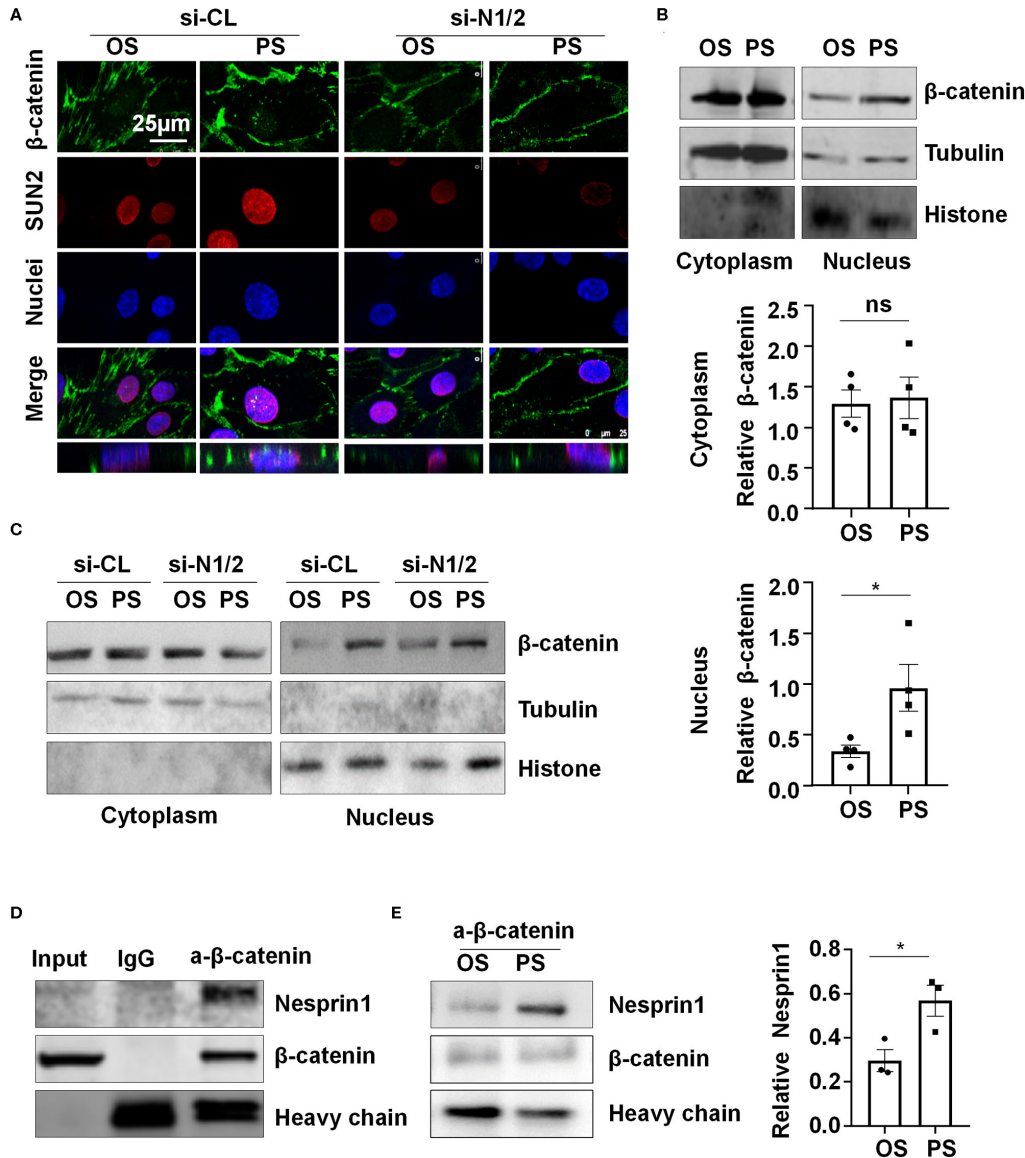
Pulsatile shear (PS) enhances the association between Nesprin1 and β-catenin to facilitate a nuclear access of β-catenin. **(A)** HUVECs were transfected with siRNAs targeting Nesprin1 and Nesprin2 (si-N1/2) (60 nmol/L) or control siRNA (si-CL) and were then exposed to PS or OS for 6 h, and the localizations of β-catenin (green) and SUN2 (red) were analyzed by immunofluorescence staining. The lower panels show 3D immunofluorescence re-construction, indicating β-catenin located mainly in cell-cell junctions and occasionally in the nuclei. **(B,C)** HUVECs with or without transfection were exposed to PS or OS and the nuclear and cytoplasmic fractions of the cells were separated to be subjected to Western blot. In **(B)**, semi-quantification of the blots are shown in the lower panels. **(D,E)** The association of Nesprin1 with β-catenin was analyzed with co-immunoprecipitation assay in static **(D)** or sheared HUVECs **(E)**. Statistical analysis of **(B,E)** were performed by unpaired *t*-test. *P < 0.05 compared with the indicated controls.

Nesprins were found to associate with β-catenin to regulate its nuclear accumulation (Zhang et al., 2016). We hypothesized that nuclear envelope-distribution of Nesprins may contribute to the entry of β-catenin into the nucleus. Using immunofluorescent staining of β-catenin, SUN2, and nuclei, followed by three-dimensional (3-D) reconstruction, we determined the cellular localization of β-catenin in si-N1/2 or si-CL transfected HUVECs under two different shear stress. Most of the β-catenin signals were detected at cell-to-cell junctions in all those cells while it is barely detectable in the nuclei in OS-exposed ECs (Figure 5A). Upon 6-h exposure to PS, the β-catenin signals in both cytoplasm and nuclei appeared, whereas to OS, the signals at the intercellular junctions did not significantly decrease. Strikingly, the cytoplasmic and nuclear accumulation of β-catenin in PS-exposed ECs was inhibited by knockdown of Nesprin1 and Nesprin2 (Figure 5A). These results indicate that PS promotes a nuclear access of β-catenin, which requires Nesprins, confirmed by cell fractionation experiments followed by Western blot assay showing that PS increased the nuclear β-catenin accumulation without affecting the amount of cytoplasmic β-catenin (including the plasms membrane and cytoplasm); the increase could be suppressed by depletion of Nesprins (Figures 5B,C).

Next, we examined how Nesprins contribute to the nuclear access of β-catenin. We measured physical interaction between Nesprin1 and β-catenin using immunoprecipitation assay in a static condition and found that an anti-β-catenin antibody could specifically precipitate Nesprin1, suggesting a constitutive association between Nesprin1 and β-catenin in ECs (Figure 5D). Six-hour PS exposure potentially enhanced the association as compared to OS (Figure 5E). Altogether, the present results suggest that PS is likely to promote association of Nesprins with β-catenin to facilitate its nuclear access.

### β-Catenin Regulates the Expression of MALAT1, ZO1, and Occludin

**Figure 6:**
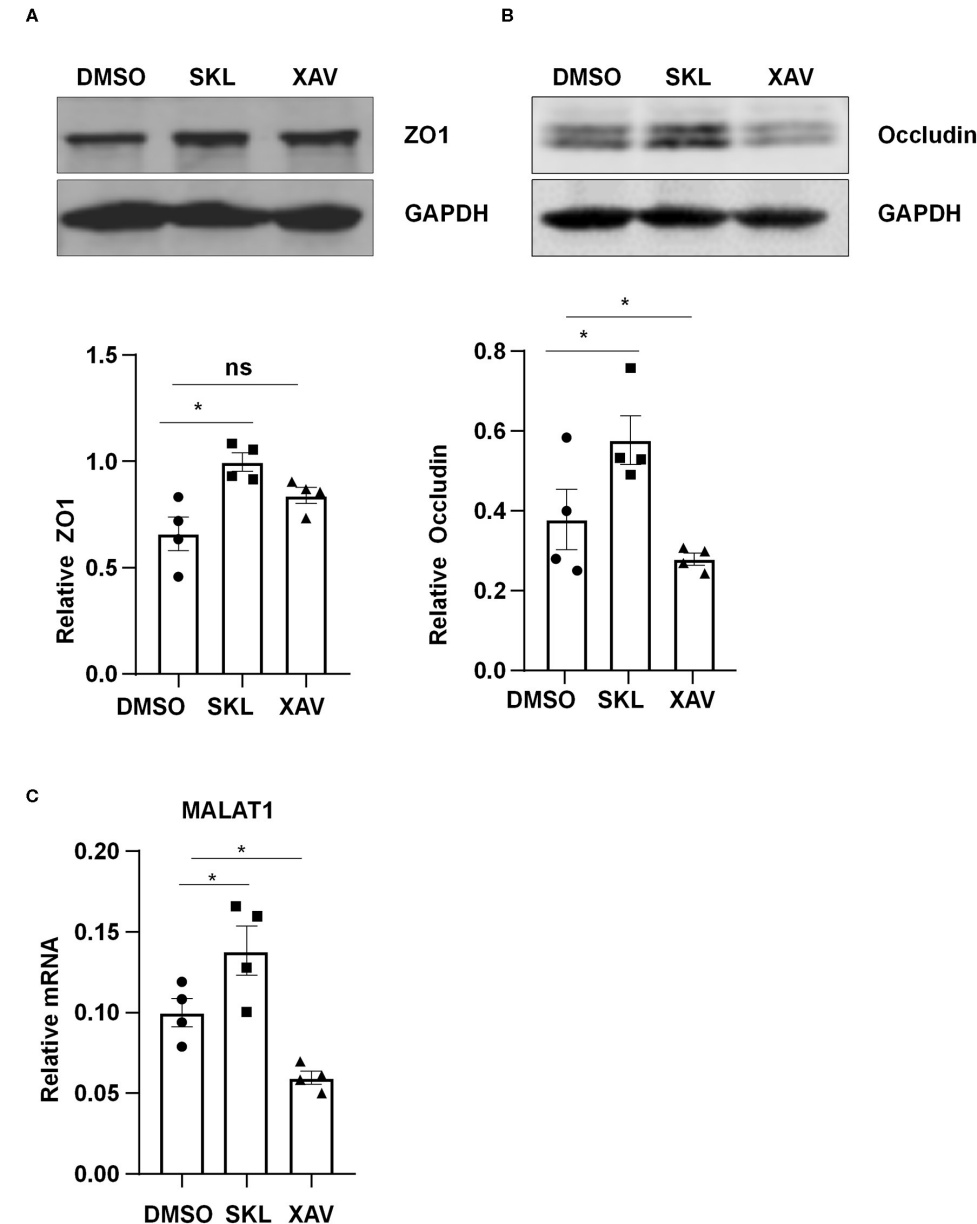
Agonist or inhibitor of the Wnt/β-catenin pathway regulate the expressions of MALAT1, ZO1, and Occludin. HUVECs were incubated with SKL2001 (30 μmol/L) and XAV939 (20 μmol/L) for 12 h, and the expressions of ZO1, Occludin, and MALAT1 were analyzed by Western blot **(A,B)** or qRT-PCR (C). In **(A,B)**, semi-quantification of the blots are shown in the lower panels. Statistical analysis of **(A–C)** were performed by One-way ANOVA. *P < 0.05 compared with the indicated controls.

Pharmacological manipulation of β-catenin activity was achieved by treating ECs with an agonist of Wnt/β-catenin pathway, SKL2001, or with a β-catenin inhibitor, XAV939. Treatment of ECs with SKL2001 at 30 μmol/L for 12 h induced the expressions of MALAT1, ZO1 and Occludin, whereas treatment with XAV939 at 20 μmol/L for 12 h decreased the expressions of Occludin and MALAT1 (Figure 6).

### β-Catenin Is Important for Maintaining Arterial Endothelial Barrier Function *in vivo*

**Figure 7:**
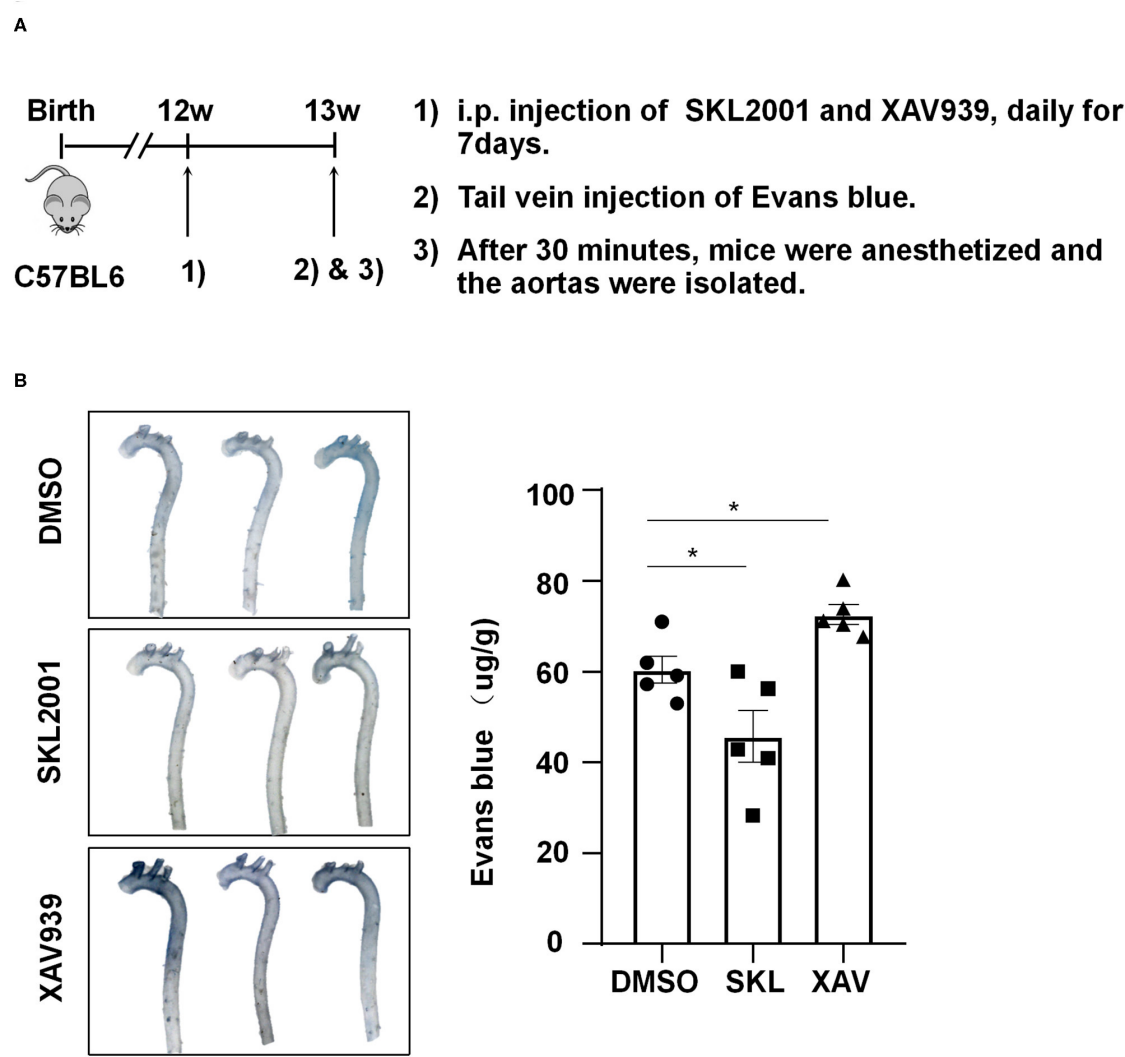
The activity of β-catenin is important for maintaining the arterial endothelial barrier function *in vivo*. **(A)** Timeline diagram of the animal experiment. **(B)** Mice were injected intraperitoneally with SKL2001 (6 mg/kg in 100 ul of DMSO), XAV939 (4 mg/kg in 100 ul of DMSO) or DMSO (100 ul) daily for 7 days. The arterial endothelial permeability to Evans blue dye was assessed. Each symbol represents one animal. One-way ANOVA, *P < 0.05 compared with the indicated controls.

To demonstrate the *in vivo* relevance of the *in vitro* findings, C57BL/6 mice aged 12 weeks were intraperitoneally injected with SKL2001 at 6 mg/kg, XAV939 at 4 mg/kg, or DMSO daily for 7 days, and they then received tail vein injection of Evans blue (30 mg/kg). Half hour after Evans blue injection, mice were sacrificed and the aortas were harvested for photographing and quantification, and the results showed a reduced wall permeability to Evans blue in SKL2001-treated mice whereas an enhanced permeability in XAV939-treated mice (Figure 7). Taken together the present results suggest that the activity of β-catenin may be required for maintaining arterial endothelial barrier function *in vivo*.

## Discussion

**Figure 8:**
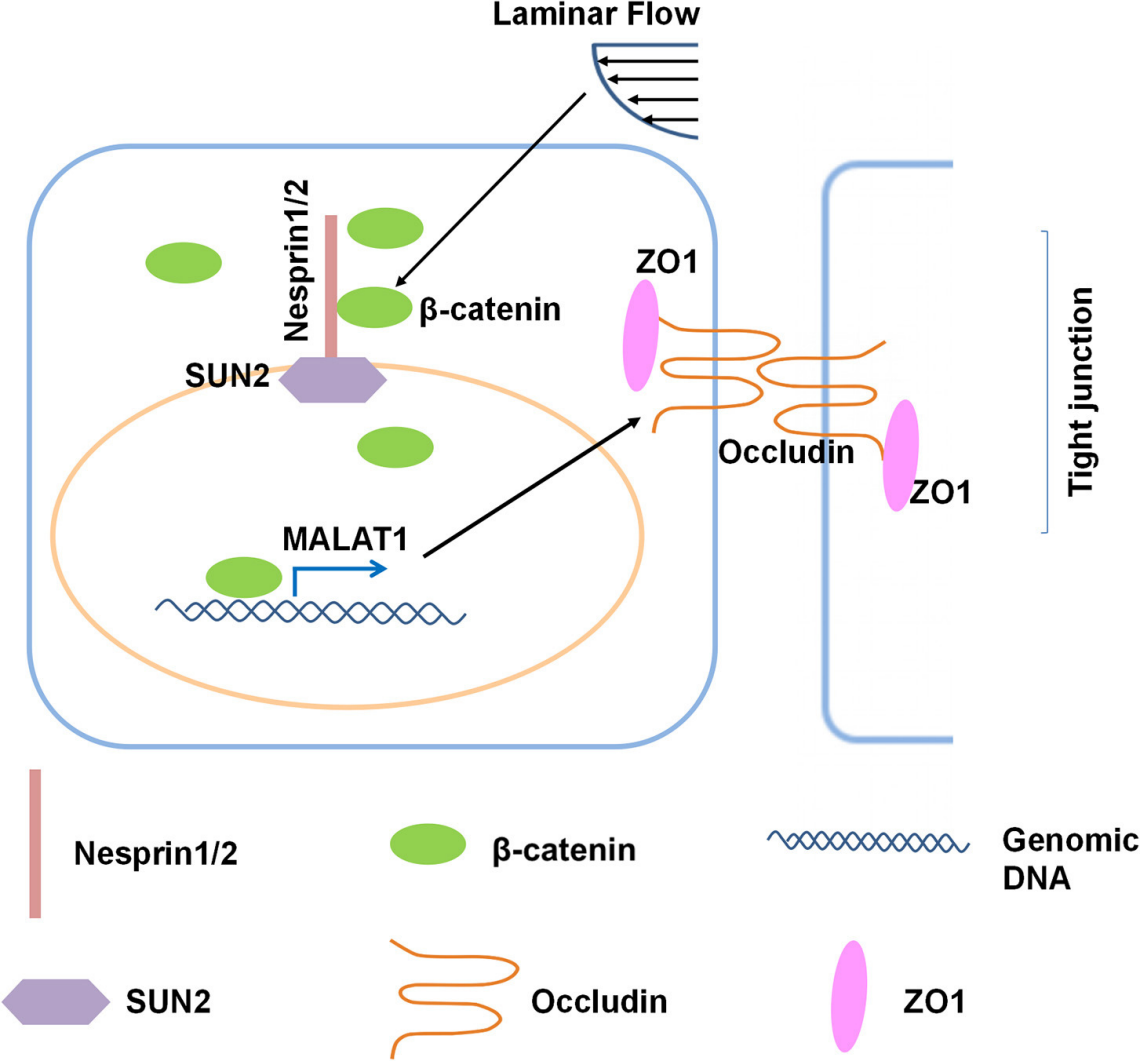
Schematic diagram of the mechanism that mediates the laminar flow-regulated endothelial tight junction and barrier function. Laminar flow enhances the physical interaction between β-catenin and Nesprin1 and the association of Nesprin1 with SUN2 at the nuclear envelopes to facilitate the nuclear translocation of β-catenin. β-catenin activates MALAT1 expression in the nucleus, thereby promoting the expressions of tight junction proteins ZO1 and Occludin.

In the present study, we investigated the role and the underlying mechanisms of atheroprotective laminar flow in regulating vascular endothelial barrier function (Figure 8). We demonstrate that laminar flow with PS stabilizes the Nesprins-mediated mechanical link between cytoplasmic skeleton with nuclear skeleton and promotes the association between Nersprin1 with β-catenin to facilitate β-catenin's nuclear access and thus to increase the expression of MALAT1 that in turn up-regulates the expressions of tight junction proteins ZO1 and Occludin to maintain endothelial integrity (Figure 8).

Increase in vascular permeability is an important pathological event during the development of atherosclerosis (Singer and Clark, 1999; Costa et al., 2007; Mazzone et al., 2009). The blood flow at the vascular branches and curvature is disturbed, resulting in uneven and irregular distribution of low wall shear stress on vascular ECs (Chiu and Chien, 2011). The combination of disturbed flow and other atherosclerotic risk factors has been suggested to jointly impairs endothelial hemostasis and barrier function (Tricot et al., 2000). Endothelial function is protected by laminar flow. Vascular ECs are connected to each other via intercellular junctional complexes including adherences junctions, tight junctions, and gap junctions (Komarova et al., 2017). Tight junctions are more developed in small arterioles, such as blood-brain barrier and inner blood-retinal barrier, thus the studies regarding the regulation of endothelial tight junctions in aortic walls are relatively limited. We observed in mouse aortas that the tight junction proteins ZO1 and Occludin at the inner curvature of AA were discontinuously distributed and that these proteins were continuously distributed and well-arranged along cell-to-cell junctions at TA (Figure 1A). We also show that the endothelial permeability to Evans blue dye at AA in mice was much higher than that at TA (Figure 1B). These findings are in line with current understanding that laminar flow is protective in maintaining endothelial integrity and barrier function. A mechanistic explanation for this phenomenon is provided by our data showing that compared to OS, prolonged PS exposure on ECs upregulates the expressions of tight junction proteins ZO1 and Occludin (Figure 1C). Thus, a critical question needs to be addressed is how PS modulates expressions of these proteins.

We provide the first line of evidence showing PS induces the expression of MALAT1 (Figure 2A), an endothelial highly expressed lncRNA that regulates ECs proliferation, migration and vessel growth (Michalik et al., 2014). It is also important to note that MALAT1 was shown before to positively regulate the expressions of ZO1 and Occludin, probably through its modulation on the activity of transcription factor CREB or nuclear factor YA (Ma et al., 2016; Ruan et al., 2019). We verified the function of MALAT1 in cultured human ECs, and demonstrated that MALAT1 is required for the expressions of ZO1 and Occludin both in static and sheared conditions (Figures 2B–F). Our results reveal that the PS-upregulated expressions of ZO1 and Occludin are MALAT1-dependent (Figure 2F).

Mechanical forces both outside and inside the cell can be transduced into biomechanical activities to direct cellular function and behavior, a process called mechanotransduction. Mechanotransduction of fluid shear stress in ECs requires several sequential steps, including physical deformation of the cell surface, intracellular stress transmission, mechanical-to-biomechanical conversion, and the downstream biochemical signaling (Davies, 2009). Although the LINC complexes have been shown to play a role in regulating the intracellular stress transmission of mechanotransduction in ECs (Morgan et al., 2011), their involvement in mediating fluid shear stress-regulated endothelial functions is rarely documented. Our study reveals that the LINC proteins, Nesprins1 and Nesprin2, are required for the expression of MALAT1 and consequently required for the expressions of ZO1 and Occludin both in static or sheared ECs (Figure 3), strongly suggesting the importance of Nesprins in mediating mechanotransduction of fluid shear stress on. The way that Nesprins respond to shear stress is likely through locating at the nuclear envelope and forming complexes with β-catenin to facilitate the nuclear access of β-catenin (Figure 5), as evidenced by the immunoprecipitation assay showing direct interaction between Nesprin1 and β-catenin (Figures 5D,E) and that knockdown of Nesprin1 and Nesprin2 inhibited PS-induced nuclear accumulation of β-catenin (Figures 5A,C). Previous studies showed that depletion of Nesprin2 or SUN1 and SUN2 could disrupt the mechanical forces-induced β-catenin trafficking into nuclei in mesenchymal stem cells and epithelial cells (Neumann et al., 2010; Uzer et al., 2018). Together with our present findings indicate that mechanotransduction through LINC is important in the regulation of cellular activity, in particular in regulating β-catenin activation. However, our study does not elucidate that how mechanical force is transmitted to the LINC complexes. Tzima et al. (2005) identified adherences junctions (VE-cadherin) and junctional adhesion molecules (PECAM-1) mechanosensory complexes that mediate the EC response to shear stress. Mechanosensing via junction proteins might also be required for the shear stress-regulated tight junctions, which needs future exploration.

β-catenin is an evolutionarily conserved multifunctional protein that exerts a crucial role in multitude of developmental and homeostatic processes (Valenta et al., 2012). It contains two pools: the cytoplasmic pool that associates with VE-cadherin to form cadherin-based adherences junctions, and the cytoplasmic/nuclear pool acting as a nuclear factor of the canonical Wnt signaling in the nucleus (Mohammed et al., 2019). Considerable evidence suggests a role of the β-catenin signaling in response to fluid shear stress with distinct flow patterns. Kamel et al. (2010) showed that PS at 16 ± 0.6 dynes/cm^2^ induced β-catenin nuclear translocation in osteocytic, osteoblastic and primary neonatal calvarial cells. Chen et al. (2019) indicated a nuclear localization of β-catenin in mesenchymal stem cells in response to laminar shear at 0.5 dynes/cm^2^. In vascular ECs, compared to laminar shear at 4 dynes/cm^2^, laminar shear at 20 dynes/cm^2^ increased the nuclear accumulation of β-catenin (Mohammed et al., 2019). Our data (Figures 5A–C) are consistent with the above studies and we demonstrate the ability of β-catenin to respond to fluid shear stress in vascular ECs. The functional consequence of β-catenin activation in ECs was further verified by the results showing that activation or inhibition of β-catenin could alter the expressions of MALAT1, ZO1 and Occludin (Figure 6).

While the effects of β-catenin agonist SKL2001 and inhibitor XAV939 have been extensively studied in cancers, tumor cell lines, and osteoclasts (Ohashi et al., 2017; Jie et al., 2019; Yu et al., 2019; Zhao et al., 2019), their potential in the treatment of cardiovascular diseases related to endothelial dysfunction is unclear. (Jean LeBlanc et al., 2019) reported that XAV939 aggravated blood-brain barrier breakdown and increased hemorrhagic transformation incidence. Another interesting finding of our study is the regulation of aortic endothelial permeability and barrier function by SKL2001 and XAV939. We demonstrated *in vivo* that systemic activation or inhibition of Wnt/β-catenin signaling results in beneficial or detrimental outcomes in the mouse aortas (Figure 7). Although we are aware of several limitations of the present study, including lack of endothelial specific depletion of β-catenin *in vivo* to confirm the role of endothelial β-catenin in regulating endothelial permeability. It is important to recognize the utilization of SKL2001 and XAV939 in this study might facilitate further evaluation of pharmacological intervention of endothelial function during the pathological development of atherosclerosis and offer novel prevention and therapeutic targets against atherosclerotic diseases.

## Data Availability Statement

The original contributions presented in the study are included in the article/Supplementary Materials, further inquiries can be directed to the corresponding author/s.

## Ethics Statement

The animal study was reviewed and approved by Committee of Peking University and approved by the Ethics Committee of Peking University Health Science Center (LA2018160).

## Author Contributions

FY contributed to the design of the experiments, trial implementation, data collection, data processing, and manuscript writing. YZ, JZ, JW, ZJ, CZ, and QY contributed to the performance of the experiments and data collection. WY and WP contributed to the design of the experiments. YH provided the useful discussions and contributed to revision of the manuscript. LH contributed to the normal operation of the experimental equipment and the safety of the experimental environment. JZ supervised and guided all the steps for the development of the manuscript, contributed to the design of the experiments, treatment and interpretation of data, and manuscript writing.

## Conflict of Interest

The authors declare that the research was conducted in the absence of any commercial or financial relationships that could be construed as a potential conflict of interest.
