# Supplementary material for: Laminar Flow Protects Vascular Endothelial Tight Junctions and Barrier Function via Maintaining the Expression of Long Non-coding RNA MALAT1
Source: Front Bioeng Biotechnol. 2020 Jun 25;8:647. doi: 10.3389/fbioe.2020.00647 (PMC7330101; doi:10.3389/fbioe.2020.00647)
Supplement: Supplementary file 1 [file Table_1.DOCX]

**Table S1**

Primer Sets

Gene Names forward reverse

ZO1 5’-GTGGGTAACGCCATCCTCTG-3’ 5’- CCATTGCTGTGCTAGTGAGC-3’

Occludin 5’-AGGAGACGTCCCCAGCC-3’ 5’-GATAAACCAATCCGCTCGCC-3’

MALAT1 5’- AGTACAGCACAGTGCAGCTT-3’ 5’-CCCACCAATCCCAACCGTAA-3’ GAPDH 5’-CATACCAGGAAATGAGCTTG-3’ 5’-ATGACATCAAGAAGGTGGTG-3’ Nesprin1 5’-GGCTGAAAATCGAAGAGACG-3’ 5’-CATCTC TGTGAGCCAGACCA-3’ Nesprin2 5’-TCACAGAGCAGCAGTCAGGT-3’ 5’-GCTCACGTTGACAGAGACCA-3’

**Table S2**

si-RNA

Gene Names Gene ID sequence

SYNE1(Nesprin1) 23345 5’-CCAAACGGCUGGUGUGAUUTT-3’

5-‘GAAGAGACGUGGCGAUUGUTT-3’

5’-GCAAAGCCCUGGAUGAUAGTT-3’

5’-GAAAUUGUCCCUAUUGAUUTT-3’

SYNE2(Nesprin2) 23224 5’-CCACAGAGCUCCAAAGUAGUU-3’

5’-GAGCAAGUGUCCCAAGAUUUU-3’

MALAT1 378938 5′-GTGACTTAAACAGCTTAAATT-3’

GAPDH 2597 5’-UUCUUCGAACGUGUAACGUTT-3’
