# Supplementary material for: Laminar Flow Protects Vascular Endothelial Tight Junctions and Barrier Function via Maintaining the Expression of Long Non-coding RNA MALAT1
Source: Front Bioeng Biotechnol. 2020 Jun 25;8:647. doi: 10.3389/fbioe.2020.00647 (PMC7330101; doi:10.3389/fbioe.2020.00647)
Supplement: Supplementary file 2 [file Image_1.pdf]

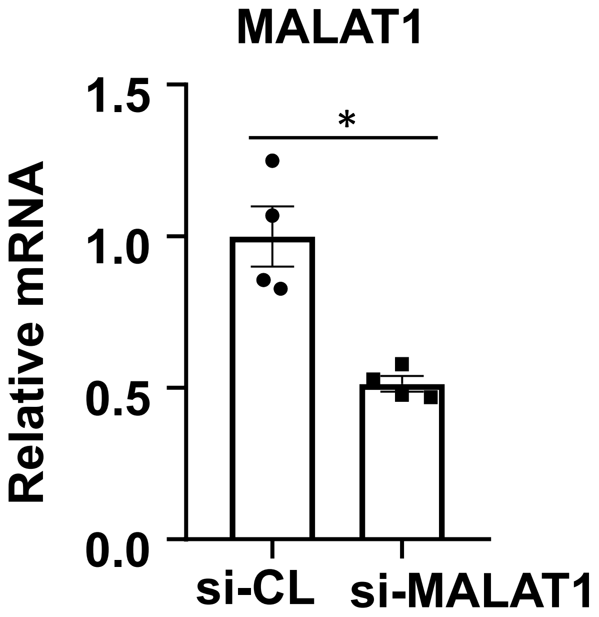

Figure S1. Efficacy of siRNA-mediated knockdown of MALAT1. ECs were transfected with siRNA targeting MALAT1, then the expression of MALAT1 was assessed by qRT-PCR assay (n = 4). Unpaired t-test . *P<0.05 compared with the indicated controls.

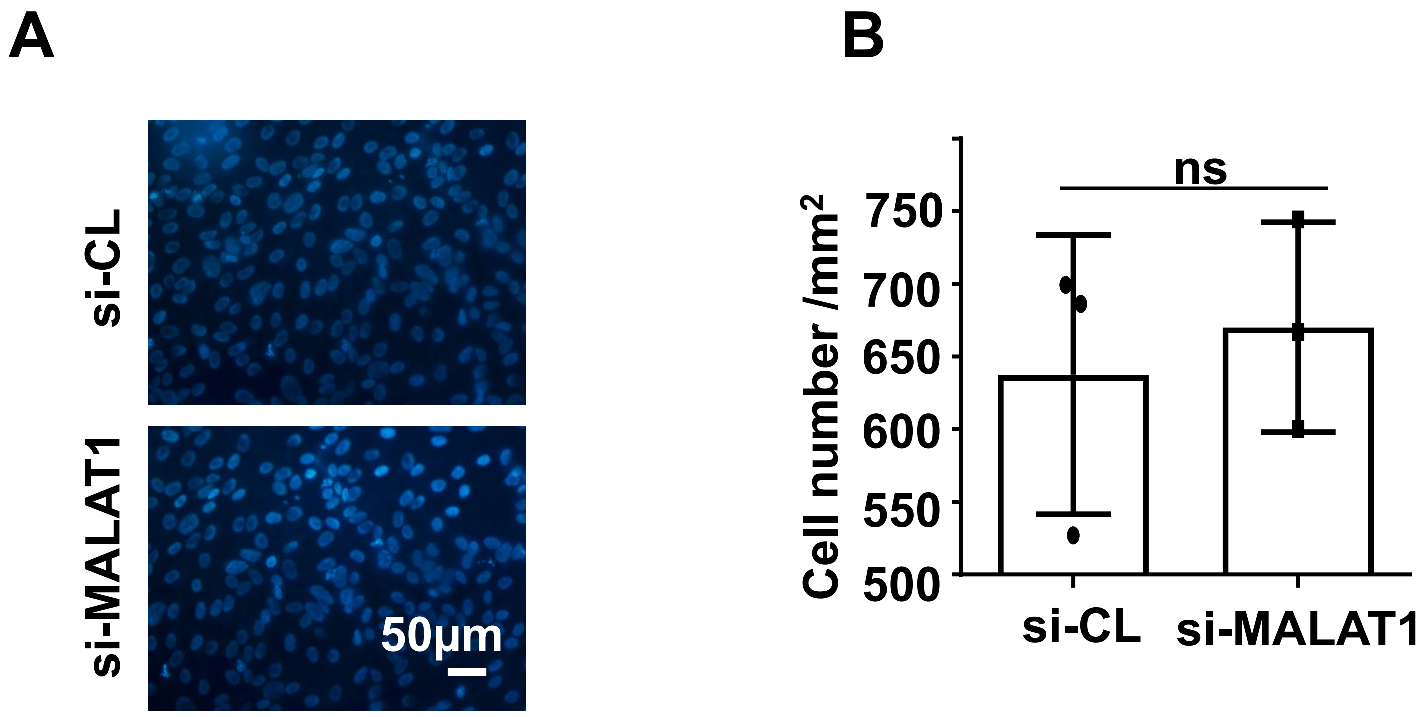

Figure S2. Analysis for cell growth in on-membrane-cultured HUVECs with MALAT1 knockdown. ECs were planted on polycarbonate membranes in transwell and were transfected with siRNAs targeting MALAT1 (si-MALAT1) or control siRNA (si-CL). The nuclei were stained with DAPI. The number of nuclei was counted under a fluorescence microscope. Unpaired t-test .

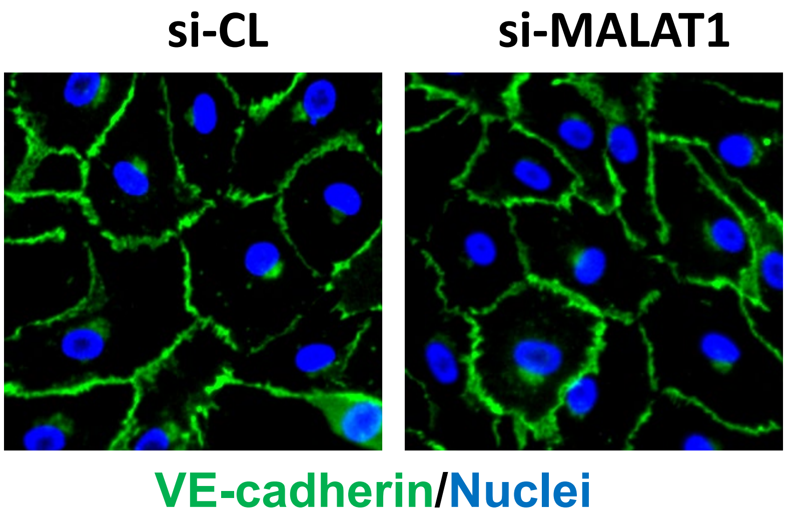

Figure S3. Immunofluorescent stating of VE-cadherin in si-CL or si-MALAT1 transfected cells. ECs were transfected with siRNAs targeting MALAT1 (si-MALAT1) or control siRNA (si-CL), and the localization of VE-cadherin (green) in the cells was analyzed by Immunofluorescence staining .

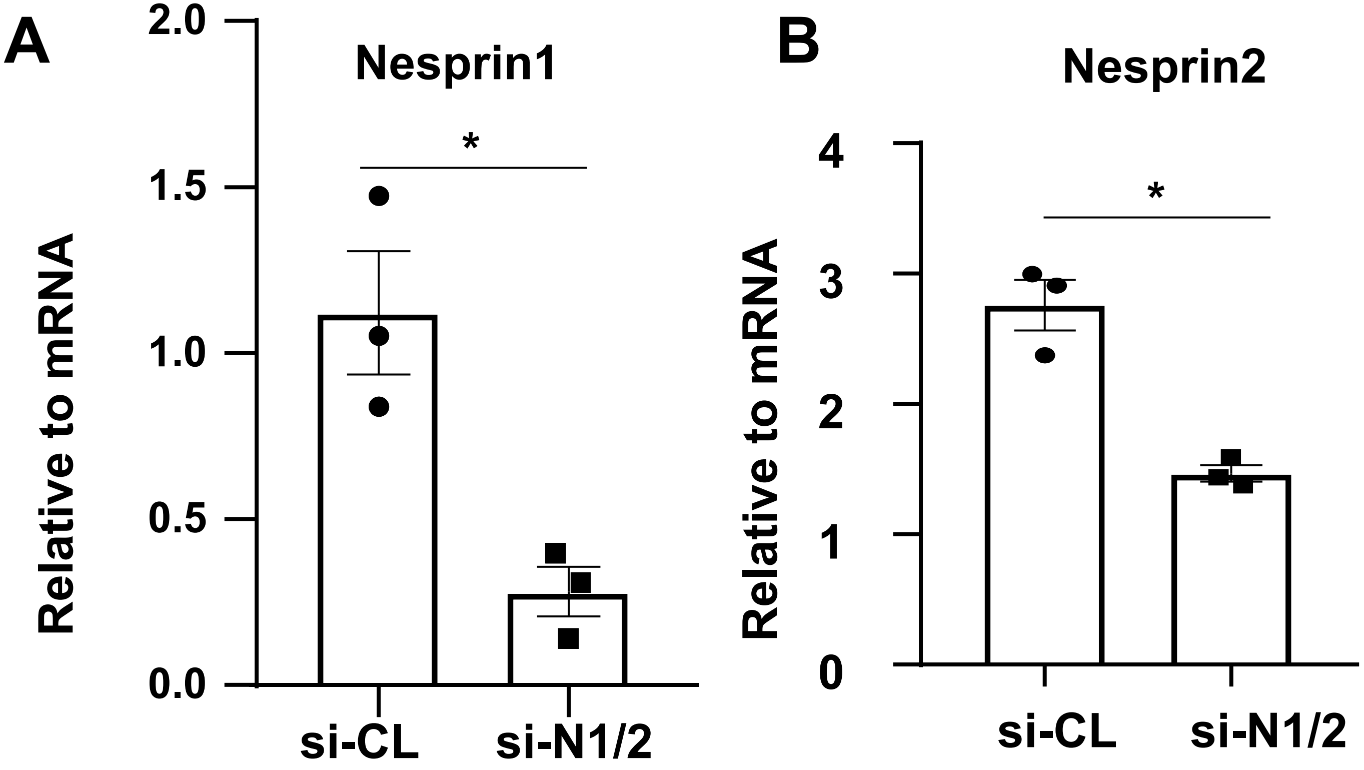

Figure S4. Efficacy of siRNA-mediated knockdown of Nesprin1 and Nesprin2. ECs were transfected with siRNA targeting Nesprin1 and Nesprin2 (si-N1/2) or control siRNAs (si-CL), and the expressions of Nesprin1 and Nesprin2 were assessed by qRT-PCR assay. Unpaired t-test . *P<0.05 compared with the indicated controls.
